# Acute Central Neuropeptide Y Administration Increases Food Intake but Does Not Affect Hepatic Very Low-Density Lipoprotein (Vldl) Production in Mice

**DOI:** 10.1371/journal.pone.0055217

**Published:** 2013-02-27

**Authors:** Janine J. Geerling, Yanan Wang, Louis M. Havekes, Johannes A. Romijn, Patrick C. N. Rensen

**Affiliations:** 1 Department of General Internal Medicine, Endocrinology and Metabolic Diseases, Leiden University Medical Center, Leiden, The Netherlands; 2 Department of Cardiology, Leiden University Medical Center, Leiden, The Netherlands; 3 Netherlands Organization for Applied Scientific Research – Metabolic Health Research, Gaubius Laboratory, Leiden, The Netherlands; 4 Department of Medicine, Academic Medical Center, University of Amsterdam, Amsterdam, The Netherlands; University of Amsterdam Academic Medical Center, The Netherlands

## Abstract

**Objective:**

Central neuropeptide Y (NPY) administration stimulates food intake in rodents. In addition, acute modulation of central NPY signaling increases hepatic production of very low-density lipoprotein (VLDL)-triglyceride (TG) in rats. As hypertriglyceridemia is an important risk factor for atherosclerosis, for which well-established mouse models are available, we set out to validate the effect of NPY on hepatic VLDL-TG production in mice, to ultimately investigate whether NPY, by increasing VLDL production, contributes to the development of atherosclerosis.

**Research Design and Methods:**

Male C57Bl/6J mice received an intracerebroventricular (i.c.v.) cannula into the lateral (LV) or third (3V) ventricle of the brain. One week later, after a 4 h fast, the animals received an intravenous (i.v.) injection of Tran^35^S (100 µCi) followed by tyloxapol (500 mg/kg body weight; BW), enabling the study of hepatic VLDL-apoB and VLDL-TG production, respectively. Immediately after the i.v. injection of tyloxapol, the animals received either an i.c.v. injection of NPY (0.2 mg/kg BW in artificial cerebrospinal fluid; aCSF), synthetic Y_1_ receptor antagonist GR231118 (0.5 mg/kg BW in aCSF) or vehicle (aCSF), or an i.v. injection of PYY_3–36_ (0.5 mg/kg BW in PBS) or vehicle (PBS).

**Results:**

Administration of NPY into both the LV and 3V increased food intake within one hour after injection (+164%, *p*<0.001 and +367%, *p*<0.001, respectively). NPY administration neither in the LV nor in the 3V affected hepatic VLDL-TG or VLDL-apoB production. Likewise, antagonizing central NPY signaling by either PYY_3–36_ or GR231118 administration did not affect hepatic VLDL production.

**Conclusion:**

In mice, as opposed to rats, acute central administration of NPY increases food intake without affecting hepatic VLDL production. These results are of great significance when extrapolating findings on the central regulation of hepatic VLDL production between species.

## Introduction

The metabolic syndrome is referred to as a cluster of physiological abnormalities correlated with obesity and type 2 diabetes mellitus [1]. Hallmarked by insulin resistance, hyperglycemia, hypertension, low high-density lipoprotein-cholesterol (HDL-C) and elevated very low-density lipoprotein-triglyceride (VLDL-TG) levels, this cluster of cardiometabolic risk factors is a strong risk factor for type 2 diabetes and cardiovascular disease [1], [2]. Furthermore, due to the strong interlinkage between its individual components, effective treatment of the metabolic syndrome has shown to be extremely challenging [2].

Obesity develops when long-term energy intake exceeds energy expenditure. The brain plays an important role in mediating energy intake, with the hypothalamus being its key regulator [3], [4]. Two major neuronal populations within the hypothalamic arcuate nucleus (ARC) exert opposing effects on energy intake. Proopio-melanocortin (POMC) neurons are activated upon food intake to exert anorectic effects by inhibiting food intake and promoting a negative energy balance. In contrast, when energy levels are low, neuropeptide Y (NPY)/Agouti-related peptide (AgRP) neurons are activated to stimulate food intake and promoting a positive energy balance [5]–[7].

The 36-amino acid peptides NPY, peptide YY (PYY) and pancreatic polypeptide, collectively called the NPY family of peptides, affect food intake by interacting with G-protein-coupled Y receptors [8], [9]. NPY is widely expressed in both the brain and the peripheral nervous system. Within the brain, NPY is highly expressed in the hypothalamus, especially in the ARC [8]. NPY-neurons co-expressing AgRP are only found in this hypothalamic nucleus, as AgRP is uniquely expressed in the ARC [10]. NPY/AgRP neurons can be activated by a diversity of signals, such as leptin and insulin [11]. Upon activation, NPY stimulates its Y receptors to activate circuits that increase food intake and fat storage [5]. Concomitantly, by antagonizing the melanocortin 3 and 4 (MC3/4) receptors in the paraventricular nucleus (PVN), AgRP prevents the catabolic drive initiated by the melanocortin system [5]. In this fashion, NPY/AgRP neurons exert a so-called double-anabolic drive.

In addition to modulation of food intake, NPY may also be involved in the regulation of lipid metabolism. A recent study in rats showed that acute modulation of central NPY signaling, either by NPY or by an Y5 receptor agonist, increased hepatic VLDL-TG production. Accordingly, central administration of a Y1 receptor antagonist decreased hepatic VLDL-TG production [12]. In mice, central NPY administration prevented the peripheral insulin-induced inhibition of glucose production by the liver, and reversed the insulin-induced inhibition of hepatic VLDL-TG production under hyperinsulinemic conditions [13]. Hypertriglyceridemia, associated with increased hepatic VLDL-TG production and/or decreased VLDL-TG clearance, is an important risk factor for cardiovascular diseases such as arterial atherosclerosis (for review [14]). Since atherosclerosis is generally studied in hyperlipidemic mice rather than in rats, we set out to validate the effect of NPY on hepatic VLDL-TG production in mice, with the ultimate goal to investigate whether NPY, by increasing VLDL-TG production, contributes to the development of atherosclerosis.

## Results

### Lateral Ventricle NPY Administration Stimulates Food Intake in Mice

**Figure 1:**
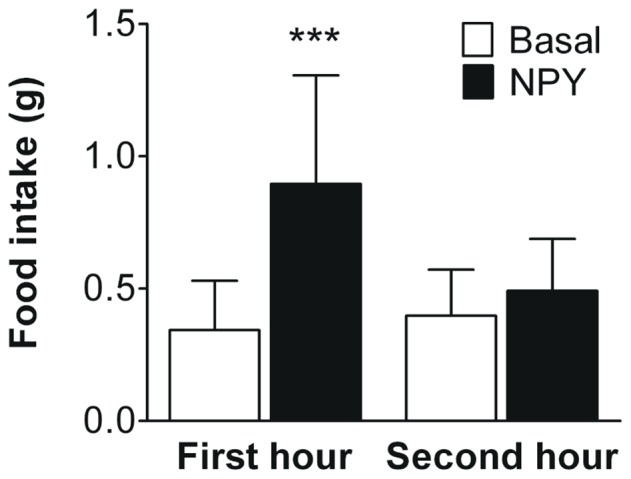
NPY administration into the lateral ventricle acutely increases food intake. NPY (0.2 mg/kg) was administered in the left lateral ventricle under light isoflurane anaesthesia, and food intake was measured for two hours, starting at 09∶00 a.m. All animals served as their own controls (basal food intake). Values are means ± SD (n = 9), ***p<0.001 compared to basal.

To verify that central administration of NPY stimulates food intake, both basal and NPY-induced food intake were assessed during two hours, starting at 09∶00 a.m. with all mice serving as their own control. Administration of NPY (0.2 mg/kg BW) in the left lateral ventricle (LV) increased food intake during the first hour after injection by +164% (0.34±0.19 vs 0.90±0.40 g, *p*<0.001, Fig. 1). Food intake during the second hour after injection was similar to the basal food intake in this specific time frame (0.40±0.17 vs 0.49±0.20 g, *n.s.*, Fig. 1).

### Lateral Ventricle NPY Administration does not Affect Hepatic VLDL Production

**Figure 2:**
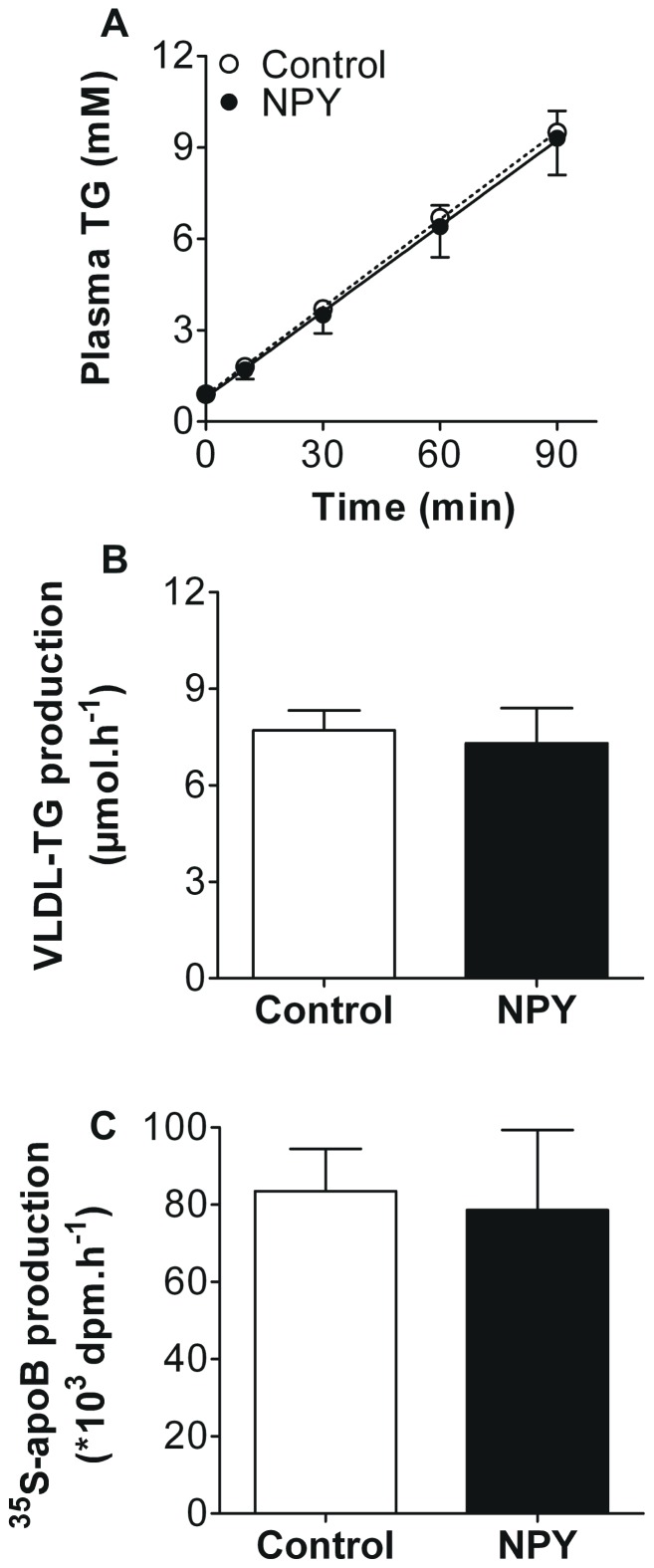
NPY administration into the lateral ventricle does not affect hepatic VLDL production in anesthetized mice. After a 4 hour fast, mice were fully anesthetized and hepatic VLDL production was assessed. Mice received an i.v. injection of Tran^35^S label (t = −30 min), followed by an injection of tyloxapol (t = 0 min), directly followed by an LV injection of NPY (0.2 mg/kg BW) or artificial cerebrospinal fluid (control). Plasma triglyceride (TG) levels were determined at indicated time points (A). VLDL-TG production rate was calculated from the slopes of the individual TG-time graphs (B). At t = 120 min, mice were exsanguinated and VLDL fractions were isolated from serum by ultracentrifugation. ^35^S-apoB production was determined by scintillation counting of the isolated VLDL fraction (C). Values are means ± SD (n = 8−10).

Next, we assessed the effects of a single injection of NPY (0.2 mg/kg BW) into the left lateral ventricle on VLDL production in 4 h-fasted anaesthetized mice. Acute central administration of NPY did not affect VLDL-TG production rate in mice (7.7±0.6 vs 7.3±1.1 µmol/h, *n.s.*, Fig. 2A, B). Accordingly, hepatic VLDL-^35^S-apoB production was also unchanged upon NPY administration (84±11 vs 79±21×10^3^ dpm/h, *n.s.*, Fig. 2C). Thus, although this dose of NPY increased food intake, it did not affect hepatic VLDL production.

Subsequently, we performed a dose-finding study to assess whether either higher or lower dosages of NPY (0.0002, 0.002, 0.02, 0.2 or 2.0 mg/kg BW) were capable of increasing hepatic VLDL-TG production. Again, we did not observe any difference between the VLDL-TG production rate in controls (6.2±0.5 µmol/h) and that in mice treated with NPY (6.9±0.1, 6.2±0.1, 6.9±0.3, 6.8±0.5 or 6.9±0.5 µmol/h at 0.0002, 0.002, 0.02, 0.2 or 2.0 mg/kg BW, respectively, *n.s.*, Fig. S1). Since the use of anesthetics theoretically could interfere with the modulation of central NPY signaling, we repeated the experiment in conscious mice. However, NPY (0.2 mg/kg BW) did not increase hepatic VLDL-TG or VLDL-apoB production in conscious mice (data not shown).

### Antagonizing Central NPY Signaling does not Affect Hepatic VLDL Production

**Figure 3:**
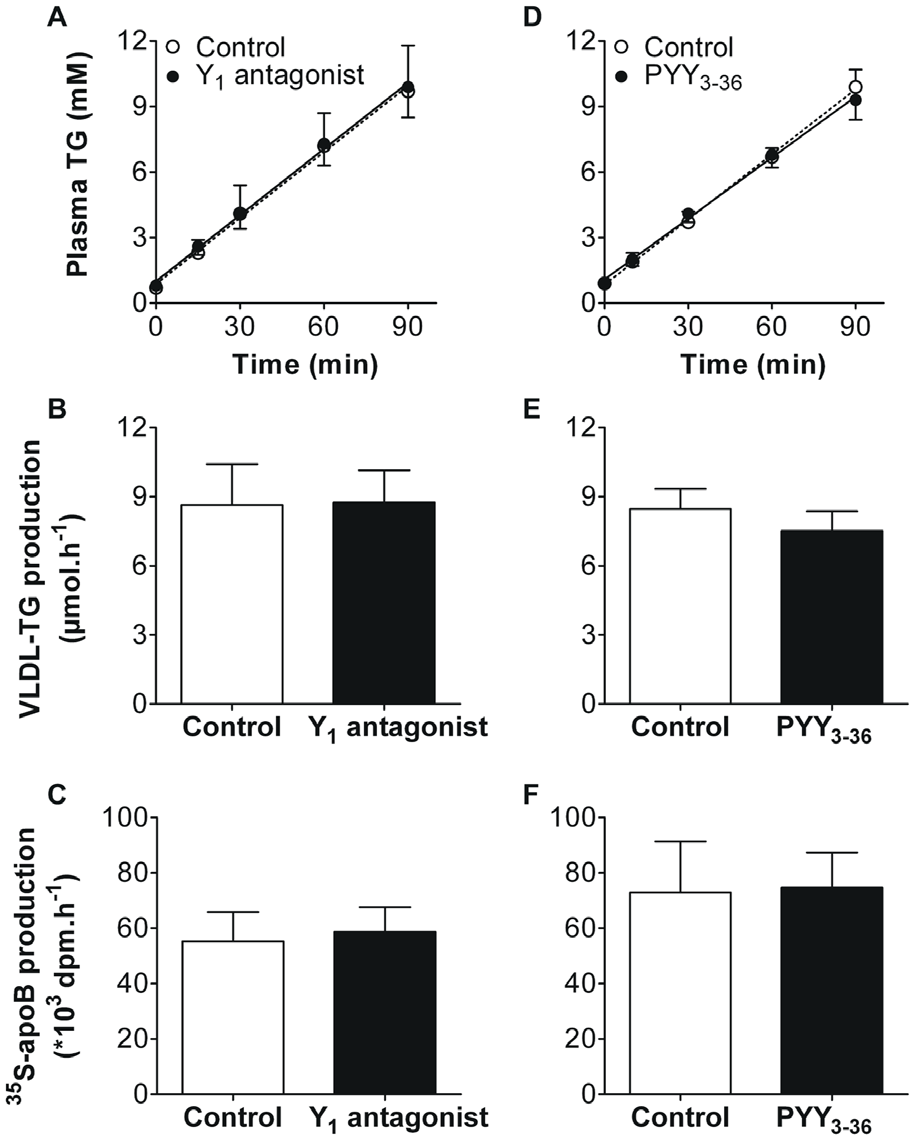
Lateral ventricle nor peripheral administration of NPY antagonists affects hepatic VLDL production in anesthetized mice. After a 4 hour fast, mice were fully anesthetized and hepatic VLDL production was assessed. Mice received an i.v. injection of Tran^35^S label (t = −30 min), followed by an injection of tyloxapol (t = 0 min), directly followed by an LV injection of GR231118 (0.5 mg/kg BW) or artificial cerebrospinal fluid (control; A–C), or by an i.v. injection of PYY_3–36_ (0.5 mg/kg BW) or PBS (control; D–F). Plasma triglyceride (TG) levels were determined at indicated time points (A+D). VLDL-TG production rate was calculated from the slopes of the individual TG-time graphs (B+E). At t = 120 min, mice were exsanguinated and VLDL fractions were isolated from serum by ultracentrifugation. ^35^S-apoB production was determined by scintillation counting of the isolated VLDL fraction (C+F). Values are means ± SD (n = 7−11).

Since other modulators of NPY signaling have previously been shown to acutely interfere with VLDL-TG production in rats [12], we next assessed the effects of PYY_3–36_ and of GR231118, a synthetic Y_1_ receptor antagonist, on hepatic VLDL-TG and VLDL-apoB production. Central administration of GR231118 did not affect the hepatic production of VLDL-TG (8.6±1.8 vs 8.7±1.4 µmol/h, *n.s.*, Fig. 3A,B) or VLDL-apoB (55±11 vs 59±9 ×10^3^ dpm/h, *n.s.*, Fig. 3C). In line with this finding, intravenous administration of PYY_3–36_, the endogenous antagonist of NPY, was also ineffective in lowering the hepatic production of VLDL-TG (8.5±0.9 vs 7.5±0.9 µmol/h, *n.s.*, Fig. 3D,E) and VLDL-apoB (73±18 vs 75±13×10^3^ dpm/h, *n.s.*, Fig. 3F).

### Third Ventricle NPY Administration Stimulates Food Intake in Mice

**Figure 4:**
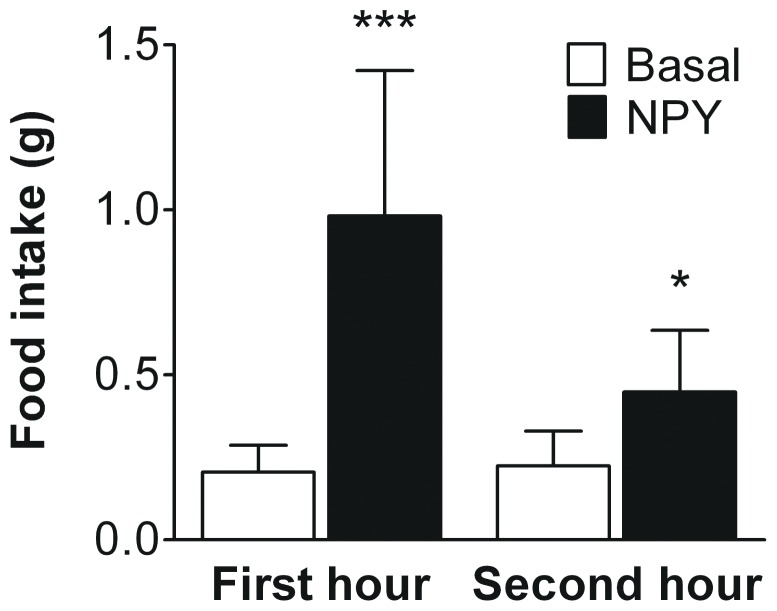
NPY administration into the third ventricle acutely increases food intake. NPY (0.2 mg/kg) was administered in the third ventricle under light isoflurane anaesthesia, and food intake was measured for two hours, starting at 09∶00 a.m. All animals served as their own controls (basal food intake). Values are means ± SD (n = 11), *p<0.05, ***p<0.001 compared to basal.

In contrast to the LV, the third ventricle (3V) is located at the base of the hypothalamus, the brain area that mediates NPY-induced feeding. To exclude that the absence of effect of modulation of central NPY signaling was due to LV versus 3V injection, we next performed 3V cannulations in mice. We first assessed the effects of 3V NPY (0.2 mg/kg BW) on food intake. NPY increased food intake during the first hour after injection by +367% (0.21±0.08 vs 0.98±0.44 g, *p*<0.001) as well as during the second hour after injection by +105% (0.22±0.11 vs 0.45±0.19, *p*<0.05) (Fig. 4).

### Third Ventricle NPY Administration does not Affect Hepatic VLDL-TG Production

**Figure 5:**
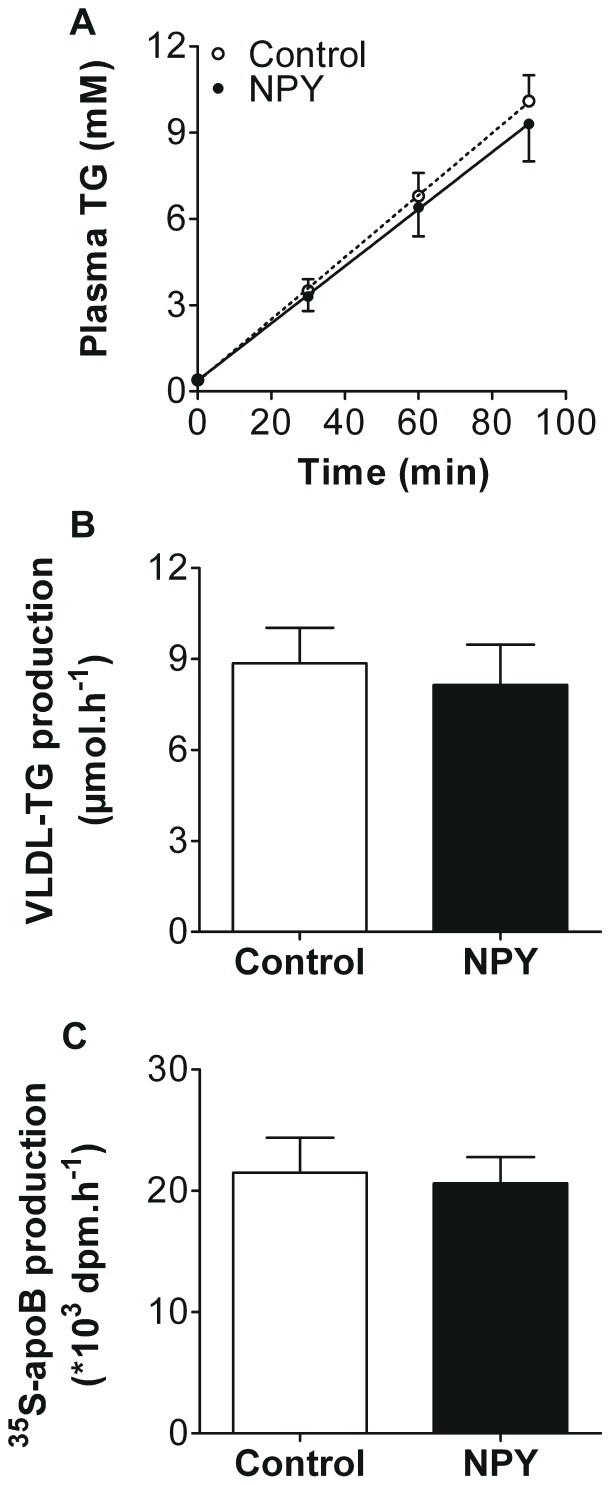
NPY administration into the third ventricle does not affect hepatic VLDL production in conscious mice. Hepatic VLDL production was assessed after a 4h-fast. Mice received an i.v. injection of Tran^35^S label (t = −30 min), followed by an injection of tyloxapol (t = 0 min), directly followed by a 3V injection of NPY (0.2 mg/kg BW) or artificial cerebrospinal fluid (control). Plasma triglyceride (TG) levels were determined at indicated time points (A). VLDL-TG production rate was calculated from the slopes of the individual TG-time graphs (B). At t = 120 min, mice were exsanguinated and VLDL fractions were isolated from serum by ultracentrifugation. ^35^S-apoB production was determined by scintillation counting of the isolated VLDL fraction (C). Values are means ± SD (n = 9−12).

Albeit that administration of NPY into the 3V also potently increased food intake, NPY (0.2 mg/kg BW) was still unable to increase hepatic VLDL production in conscious mice, as both the hepatic production rate of VLDL-TG (6.5±0.6 vs 6.0±0.9 µmol/h, *n.s.*, Fig. 5A,B) and VLDL-apoB (22±3 vs 22±2 ×10^3^ dpm/h, *n.s.*, Fig. 5C) were unchanged. Collectively, these data thus show that acute modulation of central NPY signaling does not affect hepatic VLDL production in mice.

## Discussion

Since modulation of central NPY signaling acutely increases VLDL-TG production in rats, we initially set out to investigate the acute effects of central NPY administration on VLDL-TG production in mice, ultimately aimed at investigating the contribution of central NPY, by modulating VLDL production, to the development of atherosclerosis. We confirmed that central administration of NPY acutely increases food intake in mice, similarly as in rats. In contrast to the effects in rats, central administration of a wide dose range of NPY was unable to increase VLDL-TG production in mice. Moreover, inhibition of NPY signaling by PYY_3–36_ or Y1 receptor antagonism was ineffective. In contrast to rats, in mice acute modulation of NPY signaling thus stimulates food intake but without affecting hepatic VLDL-TG production.

NPY is a well-known stimulant of food intake in both rats [15] and mice [16] and this feeding response is mediated via the hypothalamic NPY system (for review [17]). The present study confirms this effect of NPY on food intake in mice, as administration of NPY in both the LV and 3V markedly increased food intake (Fig. 1 and 4, respectively). This effect was most pronounced in the first hour after injection, which is in line with previous observations [18]. Baseline food intake was determined in conscious mice, and thus isoflurane inhalation hypothetically might have affected food intake measurements in NPY injected mice. However, in previous experiments using vehicle injections under isoflurane anesthesia, we observed an averaged food intake of 0.13 g within one hour after injection (Geerling *et al.,* unpublished data). Therefore, if any, isoflurane has an inhibiting effect on food intake and thus the increase in food intake observed in NPY injected mice can therefore not be contributed to the use of light isoflurane anesthesia. Collectively, these data indicate that NPY acutely increases food intake irrespectively of the rodent species.

Interestingly, neither LV nor 3V administration of NPY affected hepatic VLDL production in mice (Fig. 2 and 5, respectively). Furthermore, inhibition of central NPY signaling by PYY_3–36_ or the Y1 antagonist GR231118 also failed to affect VLDL production by the liver (Fig. 3). In contrast, in rats, central NPY administration was reported to acutely stimulate hepatic VLDL-TG production [12]. Bruinstroop et al [19] recently confirmed that central NPY administration acutely increases VLDL-TG production in rats. In addition, they demonstrated that the regulation of hepatic lipid production by the central NPY system in rats is guided via the sympathetic nervous system, as selective sympathetic denervation of the liver abolished the effect of central NPY administration [19].

We questioned whether differences in the experimental design between our VLDL production studies with those reported in rats [12] could have accounted for different outcomes. In mice, VLDL production experiments are commonly performed under anesthesia, whereas the studies by Stafford et al [12] and Bruinstroop et al [19] were performed in conscious rats. In theory, anesthesia could interfere with the effects of central NPY administration. For example, the μ-opioid receptor agonist fentanyl acts by inhibiting the release of multiple neurotransmitters, including the chief inhibitory transmitter gamma-aminobutyric acid (GABA) [20]. A subpopulation of NPY neurons in the ARC co-produces GABA [21]. Furthermore, NPY can act in concert with GABA to augment food intake mediated by the PVN [22]. Hence, using an inhibitor of GABA release might interfere with the effects of the centrally administered NPY. However, in the current study we show that central NPY administration also failed to increase VLDL production by the liver in conscious mice (Fig. 5). Importantly, the VLDL-TG production rates were comparable in both anesthetized and conscious mice, indicating that anesthesia did not affect baseline hepatic VLDL-TG production. Hence, the divergent regulation of hepatic VLDL production and food intake by NPY in mice cannot be explained by the use of anesthesia.

A second difference in experimental design between the rat studies and our initial setup, was the site of i.c.v. administration of NPY. Initially, we cannulated the LV in mice for obvious practical reasons, whereas Stafford et al [12] and Bruinstroop et al [19] cannulated the 3V which is more easily accessible in rats. As the third ventricle is located at the base of the hypothalamus, one could speculate that this difference in injection site might interfere with the results obtained. However, whereas 3V NPY also potently increased food intake (Fig. 4), it still did not affect hepatic VLDL-TG nor VLDL-apoB production in our hands (Fig. 5).

Interestingly, our group previously reported that LV administration of NPY was able to reverse the inhibition of hepatic VLDL-TG production in hyperinsulinemic euglycemic clamp conditions in mice [13]. This led us to conclude that insulin suppresses hepatic VLDL production at least in part by inhibiting central NPY signaling. Together with the present data, this suggests that in mice, NPY has no direct effect on hepatic VLDL production, whereas it is a downstream mediator in the suppression of hepatic lipid production by insulin.

In our study, as in previous studies [15], [16], the effects of NPY on food intake were measured in a satiated state. In contrast, hepatic VLDL production was assessed after a period of fasting, both in our study and in the previous rat studies [12], [19]. Fasting induces hypothalamic NPY mRNA expression [23]. Consequently, food intake and hepatic VLDL production were assessed during different states of endogenous NPY production, possibly leading to a different degree of sensitivity for exogenous NPY. However, the dose-finding study assessing the effects of both lower and higher dosages of NPY did not reveal any dose affecting hepatic VLDL production. Moreover, antagonizing central NPY signaling by PYY_3–36_ or an Y1 antagonist also did not affect VLDL production. Collectively, these data further support the notion that in mice, acute modulation of the central NPY system affects food intake but not hepatic VLDL production.

In addition to food intake, NPY also regulates hepatic glucose production in a similar fashion in mice and rats [13], [24]. Hence, it is tempting to speculate why NPY exerts different effects in rats versus mice on hepatic VLDL production specifically. Based on the reports of Stafford et al [12] and Bruinstroop et al [19], rats display lower basal hepatic VLDL-TG production rates when compared to those currently reported in mice. Whereas in control rats, plasma TG levels increased by ∼2 mM [12] and ∼3.5 mM [19] within one hour after tyloxapol injection, we observed that in control mice plasma TG levels are increased by ∼6 mM within the same period of time. This suggests that hepatic VLDL metabolism in itself is differentially regulated in rats versus mice.

However, the apparent species difference concerning the regulation of hepatic VLDL-TG production by NPY might also be caused by a difference in the expression of its receptor. In mammals, NPY is one of the most abundant peptides found and its receptors are widely expressed in both the central nervous system and peripheral tissues [25], [26]. Central expression of Y1–Y5 receptors is similar in rats and mice [25]. Interestingly, in addition to the Y1–Y5 receptors, mice also express the Y6 receptor. This receptor, which is a functional receptor in mice and is expressed in various brain sites including the hypothalamus [27], [28], is not expressed in rats [29]. Even though a role for the Y6 receptor in appetite regulation has been doubted [27], the exact function of the Y6 receptor remains elusive. If activation of this receptor by NPY would exert an opposing effect specifically on hepatic VLDL production, this might explain our negative findings in mice. Obviously, further investigation is needed to confirm this hypothesis. Therefore, the Y6 receptor might be an interesting target for future research investigating the role of the central NPY system in the regulation of hepatic VLDL production in mice.

Genetic association studies in humans have reported conflicting results on the role of NPY in serum TG metabolism. A polymorphism in the untranslated region between the Y1 and Y5 receptor genes was associated with lower serum TG levels in obese subjects [30]. In addition, the Leu7Pro polymorphism in the signal peptide part of the NPY gene has been linked with higher serum TG levels in preschool-aged boys [31]. However, this polymorphism was not associated with serum TG levels in female coronary heart disease patients [32]. Furthermore, studies on a variation in the 5′-flanking region of the Y2 receptor gene [33] and on the NPY signal peptide polymorphism T1128C [34] both report no association with serum TG levels. Collectively, these data emphasize the need of further research into the role of NPY in the regulation of peripheral TG metabolism. However, in light of the apparent species difference at least with respect to VLDL-TG production suggested from our study, caution should be taken when suggesting a common mechanism in humans based on findings resulting from animal studies.

In conclusion, acute central administration of NPY increases food intake without affecting hepatic VLDL production in mice, whereas NPY increases both food intake and VLDL production in rats. This apparent species difference in the effects of NPY, specifically on hepatic VLDL-TG production, is of great significance for future animal studies on the central regulation of hepatic VLDL production and underscores a general concern in animal research in view of extrapolating findings from specific animal studies to explain observations done in humans.

## Materials and Methods

### Animals

For all experiments, naive 15 weeks old male C57Bl/6J mice were used, housed in a temperature and humidity-controlled environment with free access to food and water. Experiments were performed after 4 h of fasting at 12∶00 pm with food withdrawn at 8∶00 am, unless indicated otherwise. Food intake and body weight were measured weekly during experiments. All animal experiments were approved by the Animal Ethics Committee of the Leiden University Medical Center, Leiden, The Netherlands.

### Intracerebroventricular Surgery

For i.c.v. cannula implantation, mice were anaesthetized with 0.5 mg/kg BW Medetomidine (Pfizer, Capelle a/d IJssel, The Netherlands), 5 mg/kg BW Midazolam (Roche, Mijdrecht, The Netherlands) and 0.05 mg/kg BW Fentanyl (Janssen-Cilag, Tilburg, The Netherlands) and placed in a stereotactic device (TSE systems, Homburg, Germany). A 25-gauge guide cannula was implanted into the left lateral ventricle using the following coordinates from Bregma: 1.0 mm lateral, 0.46 mm posterior and 2.2 mm ventral. For third ventricle cannulations the following coordinates from Bregma were used: 0.0 mm lateral, 1.3 mm posterior and 5.7 mm ventral. The guide cannula was secured to the skull surface with dental cement (GC Europe N.V., Leuven, Belgium) and the anesthesia was antagonized using 2.5 mg/kg BW Antipamezol (Pfizer, Capelle a/d IJssel, The Netherlands), 0.5 mg/kg BW Flumazenil (Roche, Mijdrecht, The Netherlands) and 1.2 mg/kg BW Naloxon (Orpha, Purkersdorf, Austria). Animals were single housed after the surgery.

### Food Intake Measurement

After a recovery period of at least 1 week, the mice received a pre-weighed amount of food after which basal food intake was measured for two hours, starting from 09∶00 a.m. One day later, mice received an i.c.v. injection of NPY (0.2 mg/kg in 1 µL of artificial cerebrospinal fluid, aCSF) under light isoflurane anesthesia (1.5% in air). Food was weighed before and one and two hours after waking up from the anesthesia to determine NPY-induced food intake.

### Hepatic VLDL-TG and VLDL-apoB Production

In experiments performed under complete anesthesia, 4 h fasted mice were anesthetized with 6.25 mg/kg Acepromazine (Alfasan, Woerden, The Netherlands), 6.25 mg/kg Midazolam (Roche, Mijdrecht, The Netherlands), and 0.31 mg/kg Fentanyl (Janssen-Cilag, Tilburg, The Netherlands). In other experiments, mice were awake throughout the whole experiment, except for the lateral ventricle (LV) or third ventricle (3V) injections, which were performed under light isoflurane sedation (1.5% in air).

A basal blood sample was taken from the tail tip into a chilled heparin-coated capillary (Vitrex Medical, Herlev, Denmark), and mice received an intravenous injection of 100 µl PBS containing 100 µCi Tran^35^S label (MP Biomedicals, Eindhoven, the Netherlands) via the tail vein, resulting in incorporation of ^35^S into newly produced VLDL-apolipoprotein B. After 30 min, the animals received an intravenous injection of tyloxapol (500 mg/kg body weight; Triton WR-1339, Sigma), as a 10% (w/w) solution in sterile saline, to prevent systemic lipolysis of newly secreted hepatic VLDL-TG [35].

Immediately after the tyloxapol injection, mice received an injection of either NPY (0.2 mg/kg BW, Bachem, St. Helens, UK in 1 µL aCSF) or vehicle (aCSF, 1 µL) into the lateral ventricle (LV) or third ventricle (3V). In the dose-finding study, mice received an LV injection of NPY (0.0002, 0.002, 0.02, 0.2 or 2.0 mg/kg BW in 1 µL aCSF) or vehicle. All dosages were tested once, in the number of mice indicated. In the antagonist study, mice received either an LV injection of Y1 antagonist GR231118 (0.5 mg/kg in 1 µL aCSF) or vehicle (aCSF, 1 µL) or an i.v. injection of PYY_3–36_ (0.5 mg/kg in 100 µL PBS) or vehicle (PBS, 100 µL). Both drugs were tested once, in the number of mice indicated. Blood samples were taken from the tail tip into chilled heparin-coated capillaries (Vitrex Medical, Herlev, Denmark) at the indicated time points up to 90 min after tyloxapol injection. The tubes were kept on ice after which they were centrifuged (12.000 rpm for 5 min at 4°C). Plasma TG concentration was determined using a commercially available kit according to the instructions of the manufacturer (no. 11488872**,** Roche Molecular Biochemicals, Indianapolis, IN) At 120 min, the animals were sacrificed and blood was collected by orbital puncture for isolation of VLDL by density gradient ultracentrifugation [36]. ^35^S-activity was measured in the VLDL fraction and VLDL-apoB production rate was calculated as dpm.h^−1^ [37].

### Verification of Cannula Position

After termination of mice, brains were taken out and fixed by submerging in 4% paraformaldehyde for 48 hours (Sigma-Aldrich, Zwijndrecht, the Netherlands) followed by 30% sucrose (Sigma-Aldrich, Zwijndrecht, the Netherlands) in PBS for at least 24 hours, until the brain has sank to the bottom of the container. Cannula position was verified in 30 µm thick brain cryosections mounted on microscopic slides. The sections were fixated and defatted in CARNOY solution (100% ethanol, chloroform and acetic acid in a 6∶3∶1 ratio), hydrated by descending ethanol concentrations (100-96-70%) in MilliQ (MQ) water, and a Nissl staining was performed using cresyl violet (Sigma-Aldrich, Zwijndrecht, the Netherlands): 0.9 g cresyl violet, 300 mL MQ, 2.25 mL 10% acetic acid, pH 4.5. The sections were then dehydrated in ascending ethanol concentrations (70-96-100-100%) followed by 2 times isopropanol and 2 times Histo-Clear (National diagnostics, Atlanta, USA). Cover slips were mounted using xylene, and the cannula position was verified by locating the cannula track in the tissue. When the cannula track ended within the respective ventricle, the cannula was considered to be positioned correctly. The average success rates of LV and 3V cannulation were ∼85% and ∼60% respectively.

### Statistical Analysis

Differences between two groups were determined with Mann-Whitney non-parametric tests for two independent samples. Differences between multiple groups were determined with the Kruskal-Wallis non-parametric test for *k* independent samples. When significant differences were found, the Dunn’s Multiple Comparisons test was used as a follow-up test to determine differences between two independent groups. A P-value of less than 0.05 was considered statistically significant. Data are presented as means ± SD.

## Supporting Information

Figure S1 **Higher nor lower dosages of NPY administered into the lateral ventricle affect hepatic VLDL production in anesthetized mice.** After a 4 hour fast, mice were fully anesthetized and hepatic VLDL production was assessed. Mice received an i.v. injection of Tran^35^S label (t = −30 min), followed by an injection of tyloxapol (t = 0 min), directly followed by an LV injection of NPY (0.0002, 0.002, 0.02, 0.2 or 2.0 mg/kg BW) or artificial cerebrospinal fluid (control; 0 mg/kg). Plasma triglyceride levels were determined at indicated time points (A). VLDL-TG production was calculated from the slopes of the individual TG-time graphs (B). Values are means ± SD (n = 2−5).
(TIF)
