# Supplementary figures and images for: Acute Central Neuropeptide Y Administration Increases Food Intake but Does Not Affect Hepatic Very Low-Density Lipoprotein (Vldl) Production in Mice
Source: PLoS One. 2013 Feb 27;8(2):e55217. doi: 10.1371/journal.pone.0055217 (PMC3584102; doi:10.1371/journal.pone.0055217)

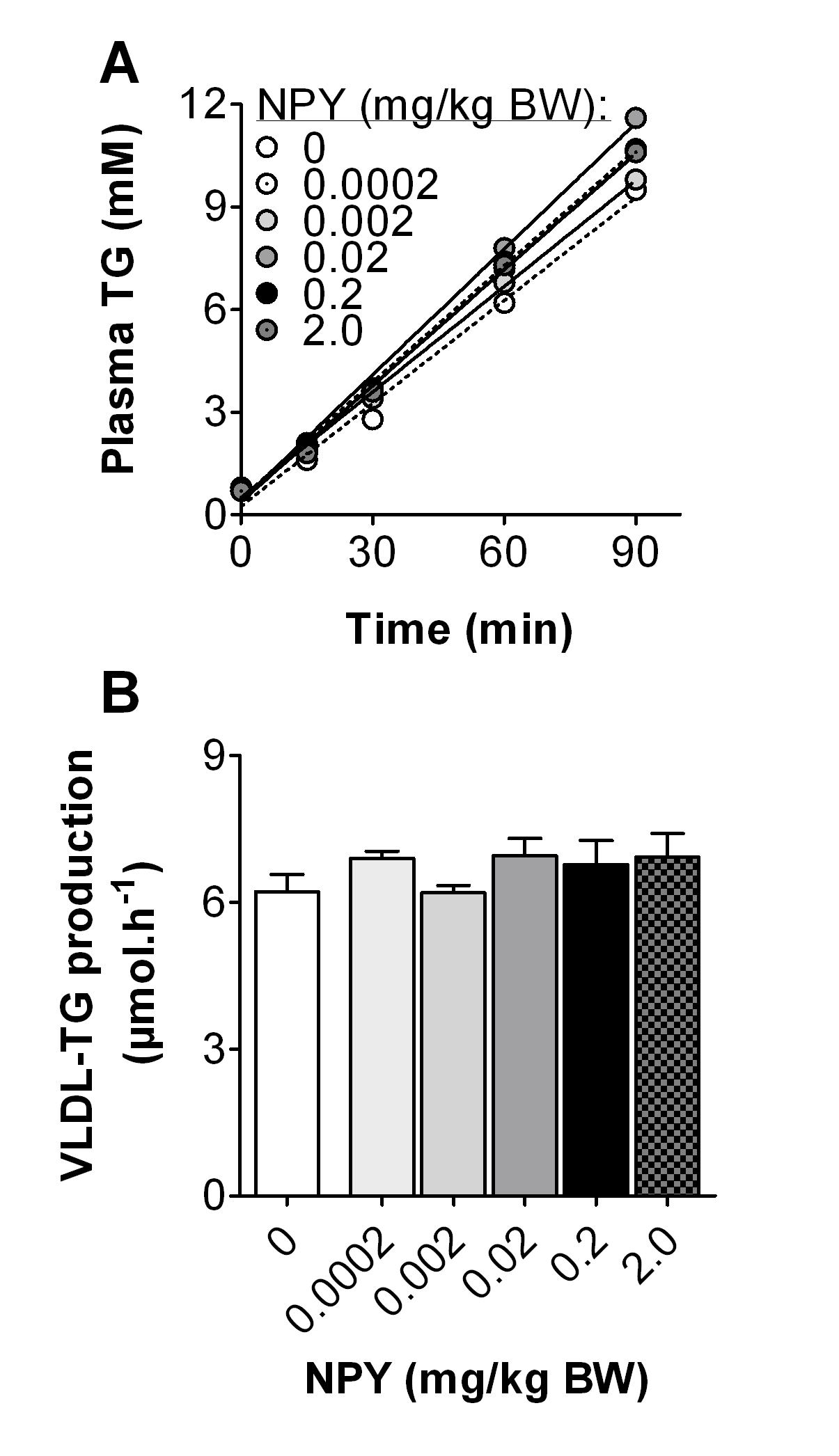

Supplement: Figure S1 — Higher nor lower dosages of NPY administered into the lateral ventricle affect hepatic VLDL production in anesthetized mice. After a 4 hour fast, mice were fully anesthetized and hepatic VLDL production was assessed. Mice received an i.v. injection of Tran35S label (t = −30 min), followed by an injection of tyloxapol (t = 0 min), directly followed by an LV injection of NPY (0.0002, 0.002, 0.02, 0.2 or 2.0 mg/kg BW) or artificial cerebrospinal fluid (control; 0 mg/kg). Plasma triglyceride levels were determined at indicated time points (A). VLDL-TG production was calculated from the slopes of the individual TG-time graphs (B). Values are means ± SD (n = 2−5). (TIF) [file pone.0055217.s001.tif]
